# Retrieving Against the Flow: Incoherence Between Optic Flow and Movement Direction Has Little Effect on Memory for Order

**DOI:** 10.3389/fnhum.2018.00102

**Published:** 2018-03-26

**Authors:** Emiliano Díez, Antonio M. Díez-Álamo, Dominika Z. Wojcik, Arthur M. Glenberg, Angel Fernandez

**Affiliations:** ^1^INICO, University of Salamanca, Salamanca, Spain; ^2^Department of Psychology, Arizona State University, Tempe, AZ, United States; ^3^Department of Psychology, University of Wisconsin-Madison, Madison, WI, United States

**Keywords:** cognitive neuroscience, memory, self-locomotion, theta rhythm, hippocampus

## Abstract

Research from multiple areas in neuroscience suggests a link between self-locomotion and memory. In two free recall experiments with adults, we looked for a link between (a) memory, and (b) the coherence of movement and optic flow. In both experiments, participants heard lists of words while on a treadmill and wearing a virtual reality (VR) headset. In the first experiment, the VR scene and treadmill were stationary during encoding. During retrieval, all participants walked forward, but the VR scene was stationary, moved forward, or moved backwards. In the second experiment, during encoding all participants walked forward and viewed a forward-moving VR scene. During retrieval, all participants continued to walk forward but the VR scene was stationary, forward-moving, or backward-moving. In neither experiment was there a significant difference in the amount recalled, or output order strategies, attributable to differences in movement conditions. Thus, any effects of movement on memory are more limited than theories of hippocampal function and theories in cognitive psychology anticipate.

## Introduction

There are multiple reasons to suspect that movement, that is, self-locomotion, should affect memory. After briefly reviewing some of these reasons, we present the results of two experiments using a virtual reality (VR) manipulation. We used VR to manipulate a component of self-locomotion that is important in both human cognitive development and the control of hippocampal processes in rat navigation: the coherence of movement and optic flow. To our surprise, we found little evidence of a link between memory and this aspect of movement.

One reason for suspecting a link between movement and memory is based on centuries-old knowledge about spatially-based mnemonics (Yates, 1966). For example, a mnemonic strategy known as the method of loci requires (a) memorization of the layout of a set of locations in space (e.g., landmarks in a town) and (b) associating the to-be-remembered material (e.g., ideas in a speech) to these locations. Later, when one wishes to recall, mentally walking through the locations in the spatial layout facilitates retrieval of the associated information. This method does not require literal movement, just the simulation, or imagination, of movement through space, and it therefore may involve imagined self-motion mechanisms that are different from perceived self-motion mechanisms (Campos et al., 2009). Nonetheless, imaginal movement may well be dependent on the functioning of the hippocampus and related neural structures that track location and movement in space (Maguire et al., 2003; Müller et al., 2017).

Second, as reviewed by Anderson et al. (2013), self-locomotion is linked to several changes in cognitive development including wariness of heights, changes in spatial coding strategies, and memory. As an example, Herbert et al. (2007) showed that experience with crawling correlates with performance in a deferred imitation task when infants are tested in new spatial contexts. Importantly for our experiments, Anderson et al. (2013) document the importance of coherence of optic flow and self-locomotion for some of the changes in cognitive development. Consider a pre-locomotor infant who is carried from one location to another. There is no opportunity for the infant to learn the correlation between optic flow and proprioception from movement. That is, because the infant is being carried, there is little relation between optic flow from movement (of the care-giver) and the infant's own motor behavior. In contrast, once the infant begins to crawl, she maintains her gaze on the goal location which induces a correlation between optic flow and proprioception from crawling. Anderson et al. (2013) refer to this as “visual proprioception,” and it is critical in several domains of control of behavior and cognitive development. For example, when visual proprioception is disrupted by suddenly moving the walls of a room, most infants will fall down. And, it is the disruption of visual proprioception at a cliff edge that Anderson et al. (2013) propose is the basis for wariness of heights. As we will see, visual proprioception also seems to play a role in the operation of rat hippocampal systems used in spatial navigation.

The strongest reasons for suspecting a link between movement and memory come from the neuropsychology literature on the role that hippocampus and associated structures play in episodic memory. Ablation of these structures creates a severe loss of memory (Squire, 1992), whereas Nobel-prize winning research has related these structures to movement, navigation, and the representation of space (O'Keefe and Nadel, 1978; Fyhn et al., 2004). Moser (2014), Schiller et al. (2015), and others have speculated that the memory and spatial functions of the hippocampus are related. And recent evidence shows hippocampus-mediated connections between gait slowing and impaired memory performance in Alzheimer's disease (Rosso et al., 2017). Although some interpret the reduced gait as a symptom of Alzheimer's disease, Anderson et al. (2013) suggest that it may also be a contributor to cognitive decline.

Our work with human participants was more directly inspired by several findings in the neuroscience literature, both with animal and human participants. Cei et al. (2014) put rats on a treadmill in a transparent cart that was pulled around a track by a toy train. The train's direction was either consistent with the animal's direction of running on the treadmill or the reverse. They found that the order of firing of hippocampal place cells (in the theta rhythm) reflected the direction in which the train was moving, indicating that movement direction is an important modulator of the hippocampal activity underlying an animal's ability to represent past, present and future locations. Note that the manipulation of the train's direction relative to the direction of self-locomotion is a manipulation of the coherence of optic flow with movement, which would be disrupted when the optic flow indicates movement in one direction and the motor proprioception indicates movement in a different direction.

Winter et al. (2015) recorded grid cell firing in rat parahippocampal cortex while the rat was engaged in self-locomotion (generating coherent visual proprioception) and while the animal was passively moved through the environment in a transparent cart (breaking the coherence). They reported that “… passive movement … abolished both velocity modulation of theta rhythmicity and grid cell firing patterns” (p. 2493). Thus, coherence between optic flow and proprioception appears to be important for spatial navigation based on the grid cell system.

In the human realm, there is empirical evidence suggesting that the entorhinal cortex is implicated in spatial processing, as shown by fMRI data reported by Nau et al. (2018), and the relation between the theta rhythm and movement has also been demonstrated (Yassa, 2018). For example, on the basis of intracranial electroencephalographic activity in the medial temporal lobe, Aghajan et al. (2017) were able to observe that theta power was significantly higher when participants moved in a real-world controlled environment than when they were stationary.

More relevant for the aim of the present study is the work by Heusser et al. (2016). They used magnetoencephalographic (MEG) recordings of gamma and theta rhythms from human adults participating in a sequence-learning memory task. They found evidence that item memory was subserved by the gamma rhythm, and item order was encoded in the theta rhythm. In their view, these results suggest an all-purpose mechanism that the brain uses to represent information about space, time, and possibly other dimensions (Aronov et al., 2017). Consistent with this argumentation, Constantinescu et al. (2016) have also proposed that the brain architecture that supports spatial navigation in human and animals can be used to organize abstract conceptual knowledge.

In view of this evidence from multiple paradigms involving animal and human research, we hypothesize that (1) the mismatch of movement and optical flow in humans should disrupt the theta rhythm in the hippocampus and related brain structures, and (2) the mismatch should thereby disrupt the processing of time-related features in memory. In other words, by disrupting the mechanisms subserving the formation and utilization of spatial representations [through the dissociation of proprioception (from self-locomotion) and optic flow], we should also disrupt memory for order.

## Experiment 1

Based on data reviewed above (e.g., Heusser et al., 2016), hippocampal mechanisms are likely to encode temporal relations even in the absence of movement. Thus, in the first experiment, participants were stationary during encoding. Then, we disrupted the coherence of optic flow and proprioception from movement (e.g., Winter et al., 2015) at retrieval.

**Figure 1:**
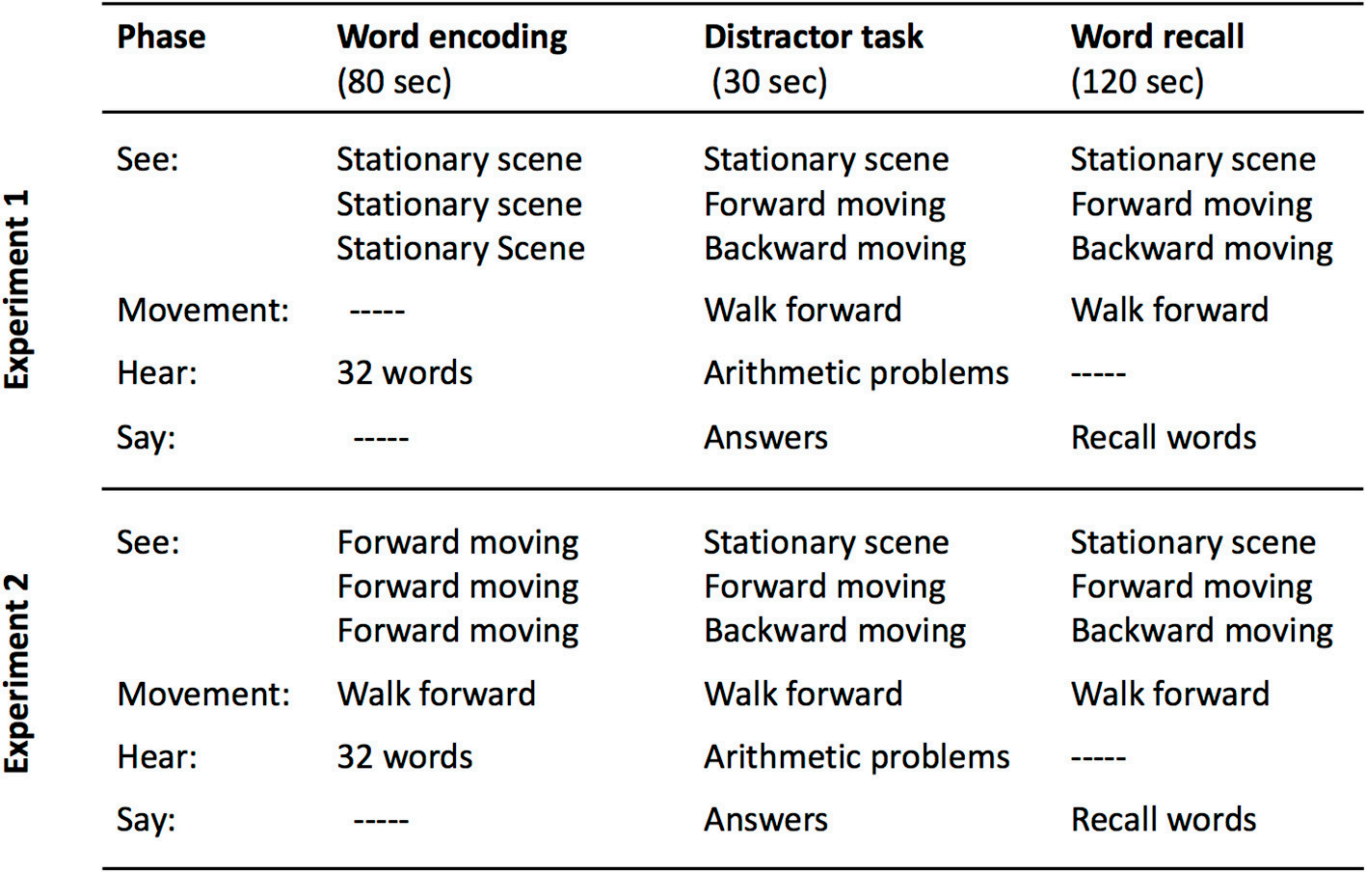
Schematic of the procedures used in Experiment 1 (top) and Experiment 2 (bottom). Note that the between-subject condition label is given in the middle column in the “See:” row.

We disrupted coherence by using VR combined with self-locomotion on a treadmill to produce a human analog to the hippocampal-activity induction scenarios in Cei et al. (2014) and in Winter et al. (2015). Adults listened to lists of words while stationary, and they were then tested for free recall while walking on a treadmill and seeing a VR landscape that was either stationary, moved appropriately with the forward direction of walking, or moved backward (see Figure 1, top). This manipulation (at retrieval) disrupts the link between self-locomotion and optic flow, the disruption should radically change place cell and grid cell firing patterns within the theta rhythm (Winter et al., 2015), and this in turn should disrupt order memory (Heusser et al., 2016). In this experiment (and the next), we analyzed output order, as well as amount of recall, and semantic and phonological retrieval strategies.

### Materials and methods

This study (and Experiment 2) was carried out in accordance with the recommendations of the Committee of Bioethics at the University of Salamanca. All subjects gave written informed consent in accordance with the Declaration of Helsinki. The protocol was approved by the Committee of Bioethics at the University of Salamanca.

In Experiment 1, the participants (*n* = 60; 21 female, 38 male, and one who did not specify gender) were native Spanish speakers between the ages of 18 and 42 years (*M* = 23.33, *SD* = 4.75). They stood on the treadmill while wearing earphones and a VR headset displaying a stationary, desert-like scene (see Supplementary Figure 1). They listened to four lists of 32 words, one word every 2.5 s. The words were 138 two-syllable Spanish nouns, randomly selected from among words with a printed frequency of 30 or more per million in the written and web-tokens database of ESPAL (Duchon et al., 2013). The words were between 3 and 7 characters long, and they ranged in frequency between 30 and 809 per million (*M* = 124.80; *SD* = 147.14). A practice list used in familiarizing participants with their task at the beginning of the session was comprised of ten words, and the other 128 words were used as to-be-remembered items in the four experimental lists presented to the participants. All the words were digitally prerecorded for auditory presentation and randomized for each participant into lists.

The last word of each list was followed by 30 s of heard arithmetic problems, and the participants spoke the numerical answers. At the beginning of this distractor task, the treadmill was started and reached a speed of 3 km/h, and the participants began to walk. For one-third of the participants (Stationary), the VR display continued to show the same static scene as during encoding; for one-third (Forward), the VR display depicted movement through the scene at a rate and direction approximating that of the participant's walking; and for one-third (Backward), the VR display depicted movement as if walking backwards through the scene, although all participants were walking forward on the treadmill. Immediately following the distractor task (and with no change in the treadmill or VR display), the participants were given 2 min to recall as many words as possible.

The desert scene used for the VR environment was not devoid of landmarks. For example, as the scene progressed, one approaches and passes palm trees, cacti, pyramids, etc. (see Supplemental Materials for a video clip). Nonetheless, as noted above, we did not control for the presentation of specific words along with specific landmarks.

### Results

**Figure 2:**
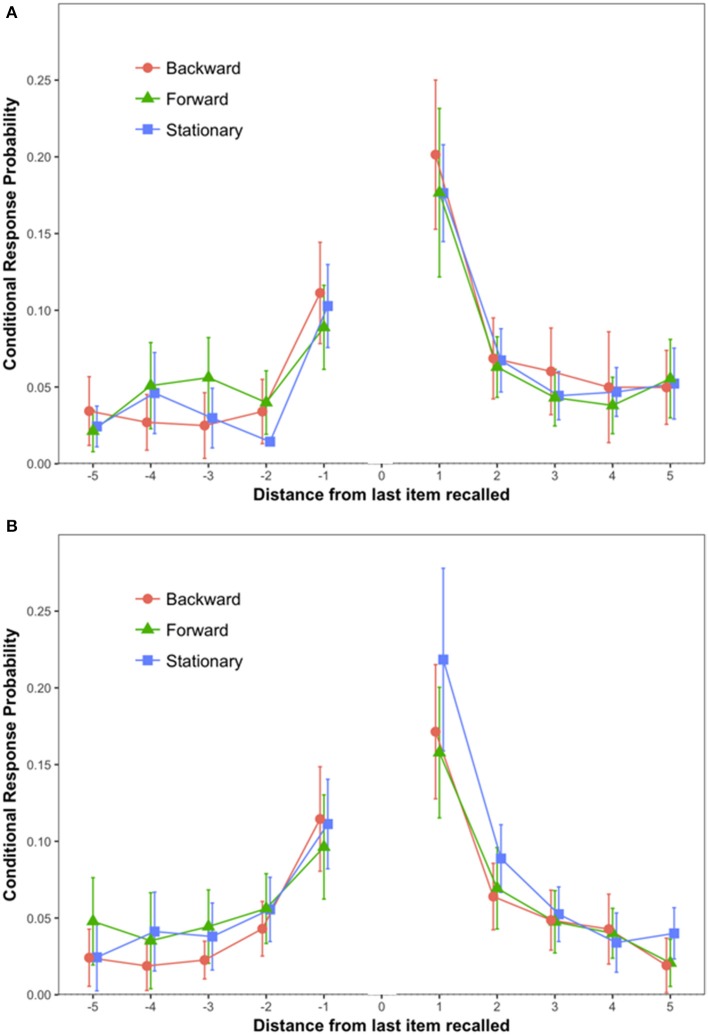
Conditional response probability by Lag and Scene Condition. The error bars are 95% confidence intervals calculated as recommended in Morey (2008). **(A)** Depicts data from Experiment 1; **(B)** depicts data from Experiment 2.

The primary dependent variable (see Figure 2A) was conditional response probability as introduced by Kahana (1996). For each pair of words recalled, we calculated the distance between the words on the input list, with positive numbers indicating that the second word was presented after the first, that is, forward recall, and negative numbers indicating backward recall. Our results replicated findings by Kahana: The most likely pairs were from adjacent input positions (lags 1 and −1), and conditional probability of forward recall (lag 1) exceeded that of backward recall (lag −1). A 3 (Scene Condition: Stationary vs. Forward vs. Backward) × 2 (Lag: 1 vs. −1) mixed ANOVA on conditional response probabilities (CRP) revealed a statistically significant effect of Lag {*F*_(1, 57)_ = 41.72, *MSE* = 0.01, *p* < 0.0001, $\eta_{p}^{2}$ = 0.42, 90% CI [0.26, 0.54]}. Tukey's test showed that CRPs for Lag 1 (*M* = 0.18; *SEM* = 0.01) were higher than CRPs for lag −1 (*M* = 0.10; *SEM* = 0.07) (*p* < 0.001).

The hypothesis of a link between coherence of self-locomotion and memory predicts an interaction between Scene Condition and Lag. That is, the Scene Condition should affect the order in which place cells and grid cells fire, and that should affect output order. However, the interaction was not significant (*F* < 1).

To determine if the data were more consistent with the null hypotheses or with the alternatives, we used Bayesian analyses computed with the anovaBF function from the BayesFactor package in R (Rouder et al., 2017). We used default priors for alternative hypotheses in factorial designs and pairwise comparisons, 100,000 iterations for convergence, and which Models = “top” option. This option compares a model that includes all of the effects in the design against a model that excludes one specific effect, with each particular effect considered in turn. If the model excluding the effect of interest is preferred over the full model, it can be taken as evidence favoring a null effect. However, if the full model is favored, then there is evidence in support of the selected effect. The Bayes Factor function produced a Bayes factor (BF) for each effect, with Bayes factors of 3:1 or greater considered as positive support for an outcome, and a Bayes factors of 20:1 or more indicating strong support (Raftery, 1995). We use BF_10_ to report evidence in favor of the alternative hypothesis, and BF_01_ to report evidence in favor of the null hypothesis.

Bayesian analysis of the difference between lags −1 and 1 strongly favored the alternative, with BF_10_ > two million to one. But the differences among the Forward, Backward, and Stationary conditions were quite small, and for the interaction of Scene Condition and Lag, the Bayesian analysis favored the null hypothesis with BF_01_ = 6.6:1.

In a separate analysis, there was no effect of Scene Condition on overall amount recalled, *F*_(2, 57)_ = 1.65, *p* = 0.20. The Bayesian analysis showed weak support for the null hypothesis with BF_01_ = 2.21:1.

We also looked at clustering by semantic and phonological measures, as strategic processing could mask the effects of the motion manipulation. First, a semantic clustering measure (SC) was calculated for each recall output stream (trial), as a vector of semantic relatedness values (SR) between adjacent words. The SR was calculated from the EN_100k_lsa corpus, an LSA (latent semantic analysis) space reduced to 300 dimensions and derived from a ~2 billion-word corpus, which was created by concatenating the British National Corpus, the ukWaC corpus and a 2009 Wikipedia dump (Repository for Semantic Spaces, 2017). All calculations were conducted with the LSAfun package for R (Günther et al., 2015). For each participant, the mean of semantic relatedness vector scores across trials was calculated, and then those scores were analyzed by way of a one-factor ANOVA (Scene Condition: Stationary, Forward or Backward). The analysis revealed a non-significant effect {*F*_(2, 57)_ = 1.41, *MSE* = 0.00, *p* = 0.25, $\eta_{p}^{2}$ = 0.05, 90% CI [0, 0.14]}. Thus, the degree of semantic clustering did not seem to vary with Scene Condition.

Second, a phonological clustering (PC) measure was calculated for each trial by way of the Damerau–Levenshtein distance, a string metric for measuring the edit distance between two sequences defined as the minimum number of operations (insertions, deletions or substitutions of a single character, or transposition of two adjacent characters) required to change one word into the other. All calculations were conducted with the *stringdist* package for R (van der Loo, 2014). For each participant, the mean of the trial phonological relatedness vector scores across trials was calculated and, then, those scores were analyzed by way of a one-factor ANOVA. The analysis revealed a non-significant effect of Scene Condition, {*F*_(2, 57)_ = 0.11, *MSE* = 0.04, *p* = 0.90, $\eta_{p}^{2}$ = 0.004, 90% CI [0, 0.02]}. Thus, the degree of PC did not seem to vary with Scene Condition.

## Experiment 2

In Experiment 2 we test another variant of the hypothesis, considering that perhaps hippocampal mechanisms are primed by literal movement during encoding. Then, memories encoded with these mechanisms can be disrupted by dissociating optic flow and proprioception at retrieval. Thus, during encoding, participants walked on the treadmill while viewing the Forward VR scene. During retrieval, the participants continued to walk forward, but the scene was manipulated (see Figure 1, bottom).

### Methods

In Experiment 2, the participants (*n* = 60, 42 female) were native Spanish speakers, between 19 and 45 years of age (*M* = 20.42, *SD* = 3.62). There was no overlap with the participants in Experiment 1. The participants listened to the four lists while walking forward on the treadmill with the Forward VR. After listening to the words, all participants continued to walk forward, although the VR showed the Stationary, Forward, or Backward scenes to different participants beginning during the distractor task and continuing throughout the recall period (see Figure 1, bottom).

### Results

The conditional response probabilities are shown in Figure 2B. A 3 (Scene Condition: Stationary vs. Forward vs. Backward) × 2 (Lag: 1 vs. −1) mixed ANOVA on conditional response probabilities (CRP) revealed a statistically significant effect of Lag {*F*_(1, 57)_ = 23.38, *MSE* = 0.007, *p* < 0.0001, $\eta_{p}^{2}$ = 0.29, 90% CI [0.13, 0.42]}. Tukey's test showed that CRPs for Lag 1 (*M* = 0.18; *SEM* = 0.01) were higher than CRPs for lag −1 (*M* = 0.11; *SEM* = 0.01) (*p* < 0.001). No other source of variability reached statistical significance.

The Bayesian analysis confirmed that there was a large effect of Lag (BF_10_ > 1,000:1), but for the interaction of Scene Condition and Lag the data favored the null hypothesis (BF_01_ = 3.3:1).

As with the first experiment, Scene Condition did not affect amount recalled (*p* = 0.20, BF_01_ = 2.20:1, weakly favoring the null). Also, as in Experiment 1, semantic and PC measures were calculated and analyzed by way of one-way ANOVAs. The analyses showed no significant effects of scene condition on semantic clustering {*F*_(2, 57)_ = 0.53, *MSE* = 0.00, *p* = 0.59, $\eta_{p}^{2}$ = 0.02, 90% CI [0, 0.08]} or PC {*F*_(2, 57)_ = 0.15, *MSE* = 0.04, *p* = 0.86, $\eta_{p}^{2}$ = 0.005, 90% CI [0, 0.04]}.

## Discussion

Given the strong reasons to suspect a link between memory and the coherence of movement and optic flow, we were surprised by the findings reported here. Note that our null effects of Scene Condition are not due to low power. According to the Bayesian analyses, in both experiments the effect of Lag was overwhelming, and there was strong support for the null hypothesis of no interaction between Scene Condition and Lag [across the two experiments, the overall support for the null of no interaction is (6.1 × 3.3) around 20:1].

Clearly, these results speak against the hypothesis of a dependency between order memory and coherence of optic flow and self-locomotion. That is, although we disrupted the correlation of optic flow and proprioception, there was no effect on memory retrieval. Whether similar null results of lack of movement-flow coherence will be found on other processes (e.g., encoding) remains to be tested.

Given the reasons for suspecting a relation between movement and memory, what can explain our results? There is the possibility that our manipulations did not succeed in disrupting theta firing. But this possibility seems unlikely given the effects found by Aghajan et al. (2017) and Winter et al. (2015). Another possibility is that the hippocampal function is complexly organized. For example, Spiers et al. (2001) report that patients with a right temporal lobectomy are impaired in navigation tasks, whereas patients with left temporal lobectomy are impaired in episodic memory tasks. If hippocampal function is lateralized in this way, there would be little reason to suspect that disrupting the correlation of optic flow and proprioception would disrupt memory for order.

Of course, there are many ways to continue to look for a link between self-locomotion and memory. For example, other memory paradigms can be used (e.g., see Loeffler et al., 2017 for a procedure which used walking-backward to facilitate the processing of past-related stimuli). Perhaps the to-be-remembered materials should be related to the visual environment; perhaps a manipulation of coherence of movement and optic flow during encoding would be more effective; perhaps effects of movement on memory are prominent during early or late development. Nonetheless, given the strong mnemonic, developmental, and neuropsychological reasons for suspecting a connection between self-locomotion and memory, the coherence of movement and optic flow affected memory surprisingly little.

## Author contributions

All authors contributed to the design of the experiments; AD-Á and DW collected the data; ED analyzed the data. All authors contributed to writing the manuscript.

### Conflict of interest statement

The authors declare that the research was conducted in the absence of any commercial or financial relationships that could be construed as a potential conflict of interest.
