# Supplementary figures and images for: Retrieving Against the Flow: Incoherence Between Optic Flow and Movement Direction Has Little Effect on Memory for Order
Source: Front Hum Neurosci. 2018 Mar 26;12:102. doi: 10.3389/fnhum.2018.00102 (PMC5879133; doi:10.3389/fnhum.2018.00102)

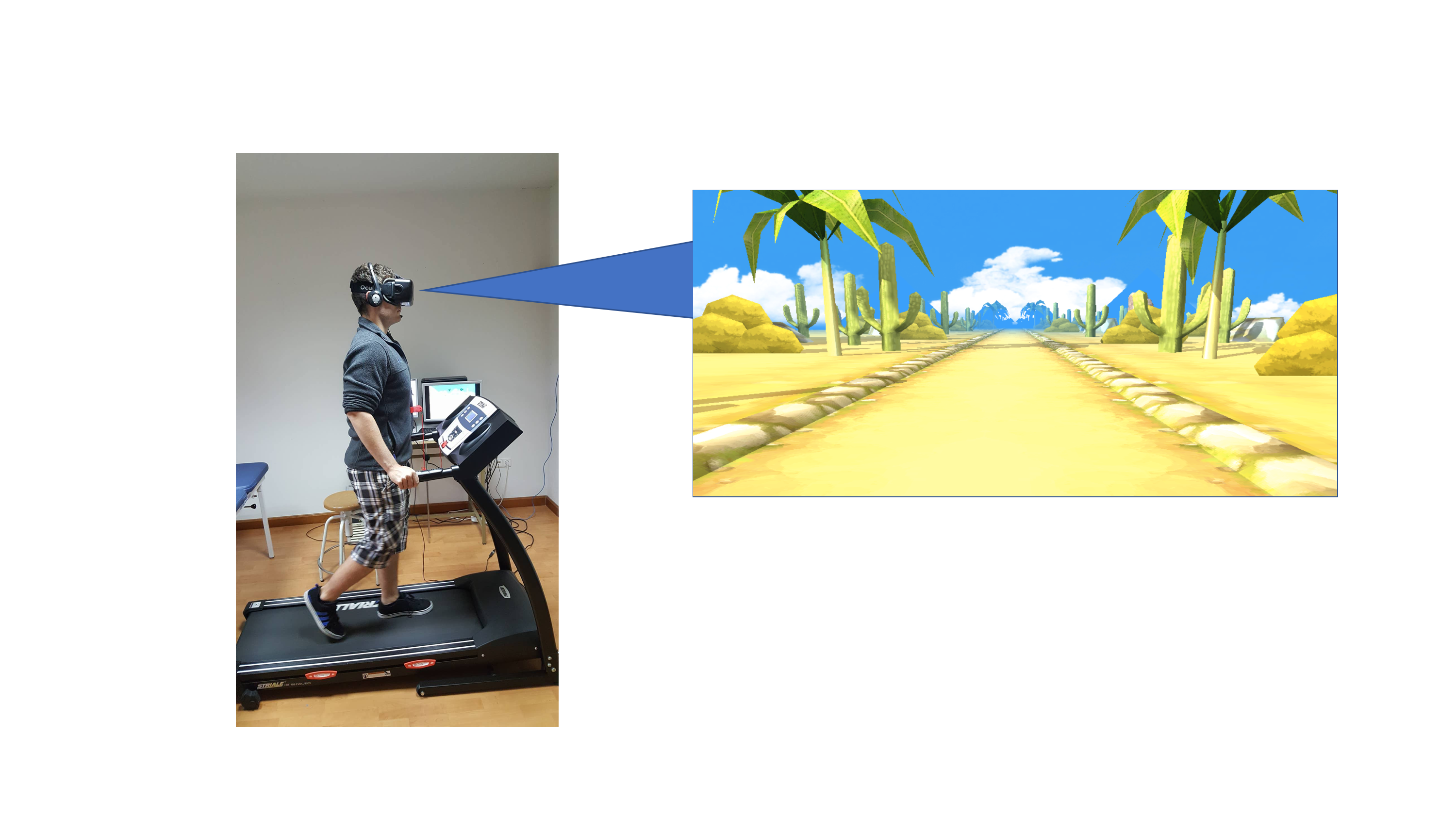

Supplement: Supplementary file 3 [file Image1.png]
